# Well-Differentiated Neuroendocrine Tumors of Lung: A Case Series Supporting the IARC/WHO Common Classification of Neuroendocrine Tumor Nomenclature and Grading

**DOI:** 10.1007/s12022-026-09914-2

**Published:** 2026-04-01

**Authors:** Eren Altun, Omid Savari, Mart Andrew Maravillas, Sylvia L. Asa

**Affiliations:** 1 Department of Pathology, University Hospitals Cleveland Medical Center, Seidman Cancer Center, Case Western Reserve University, Cleveland, OH USA; 2 Department of Pathology, University of Health Sciences, Istanbul Bagcilar Training and Research Hospital, Istanbul, Türkiye; 3 Clinical Research Biostatistician, University Hospitals Cleveland Medical Center, Cleveland, OH USA

**Keywords:** Ki67, Mitosis, Lung, Neuroendocrine neoplasm (NEN), Neuroendocrine tumor (NET), Carcinoid

## Abstract

The aim of this study was to assess whether there is sufficient evidence to recommend a change in the classification of pulmonary tumors currently classified as “carcinoid” tumors to instead apply a system similar to that used for well-differentiated gastrointestinal and pancreatic neuroendocrine tumors (NETs) using the Ki67 proliferation index (PI). We retrospectively reviewed 124 cases of pulmonary NETs (“typical carcinoids” (TC) and “atypical carcinoids” (AC)) diagnosed at the University Hospitals Cleveland Medical Center between 2019 and 2025. Demographic, clinical, and pathology data were collected, including mitotic counts, necrosis, and Ki67 PI assessed in biopsy, resection, and metastatic specimens. Tumors were graded as G1 (≤ 3%), G2 (3–20%), and G3 (> 20%) according to the 5th edition WHO criteria for gastroenteropancreatic NETs. Grading based on Ki67 PI was significantly associated with lymphatic invasion (*p* = 0.007), lymph node metastasis (*p* = 0.016), liver metastasis (*p* = 0.047), AJCC stage at diagnosis (*p* < 0.001), and patient mortality (*p* = 0.047). This approach provided better correlation than necrosis for liver metastasis and mortality, better than mitoses for all negative outcomes than other than liver metastasis, and better than TC/AC classification for mortality. Tumors classified as G3 had significantly worse patient outcomes than those classified as G1 or G2. Incorporating Ki67 PI into the classification provides clinically important information regarding tumor progression, staging, and survival. These findings support the adoption of a three-tiered Ki67-based grading system (G1–G3) for pulmonary NETs, ​​similar to gastroenteropancreatic NETs.

## Introduction

Neuroendocrine neoplasms (NENs) of the lung are heterogeneous, ranging from slowly progressing lesions with a relatively long survival time to highly aggressive, treatment-resistant tumors with a very poor prognosis. According to the World Health Organization’s (WHO) 2021 classification of thoracic tumors, pulmonary NENs account for approximately 20% of all lung malignancies and encompass both well differentiated neuroendocrine tumors (NETs) and poorly differentiated neuroendocrine carcinomas (NECs) [1]. The classification groups pulmonary NENs into four histological variants — typical carcinoid (TC), atypical carcinoid (AC), large cell neuroendocrine carcinoma (LCNEC), and small cell lung carcinoma (SCLC). The subclassification of lung carcinoids is based on the assessment of mitotic index and necrosis; AC is distinguished from TC based on histological evaluation of the mitotic count (0–1 mitoses/2 mm² for TC and ≥ 2 mitoses/2 mm² for AC) and the presence of focal or punctate necrosis and this can only be assessed in resection specimens, so biopsies are classified as “carcinoid NOS”. The importance of accurate classification is the impact on management and outcome [2], since up to 10% of patients with tumors classified as lung carcinoids develop recurrence as late as 15 years after initial surgery, highlighting the need for long-term follow-up for all patients. ACs have a higher metastatic rate and a shorter disease-free 5-year survival rate following curative resection, and surveillance varies since TCs have greater expression of somatostatin receptors (SSTRs) and higher ^68^Ga-DOTA-peptide PET/CT positivity than ACs, whereas ACs exhibit greater FDG uptake than TCs [3, 4]. However, staging based on tumor size, nodal involvement and metastasis appears to be the most significant prognostic factor associated with recurrence, regardless of histological subtype [5, 6].

NENs at other sites have been reclassified into a common classification approach that distinguishes NETs from NECs based on molecular alterations and in most sites, grades tumors into three grades (G1, G2 and G3) based on proliferative indices [7]. The traditional classification of lung NETs is unique in its conspicuous lack of a role for the Ki67 proliferation index (PI). The Ki67 stain is used to distinguish “carcinoids” from high-grade LCNEC and SCLC, and is particularly important in small biopsy samples with crush artifact [8], however, a definitive cut-off value for Ki67 in distinguishing TC from AC has not been established. While several studies have evaluated the full spectrum of lung NENs with various recommendations on how to incorporate the Ki67 PI [9–28], there is no consensus on the optimal Ki67 cutoff value or approach and Ki67 has not been incorporated into the classification. Moreover, the concept of a “grade 3 well-differentiated neuroendocrine tumor” has been discouraged in the classification of pulmonary NENs [3, 8].

Like NENs at other sites, the distinction between pulmonary NET and NEC has been shown to have a molecular basis [29]. The well differentiated tumors are characterized by a low mutational burden, and a high prevalence of mutations in chromatin remodeling and histone modification-related genes like *MEN1*, whereas high-grade NECs show inactivation of the *TP53* and *RB1* genes, along with upregulation of c-Kit, platelet-derived growth factor receptor (PDGFR), epidermal growth factor receptor (EGFR), and other tyrosine kinase receptors, and have a higher rate of chromosomal aberrations involving 3p, 17p, and other loci. Despite this, markers of molecular alterations are not recommended for the distinction of NET from NEC and there is no recognition of a highly proliferative G3 NET. Recent literature has documented the presence of lung NETs with morphological features of AC but with higher mitotic counts (> 10 mitoses/2 mm2) and/or higher Ki67 PI values, some higher than 30% [19, 30]. Such tumors are rare at primary sites but not uncommon in the metastatic setting [31]. Additional information including molecular alterations that can be insinuated by immunohistochemistry (such as loss of Rb1 and/or mutation of p53) may be more useful to distinguish between highly proliferative pulmonary NET and LCNEC.

The term ‘carcinoid’ is problematic for two reasons: firstly, it means ’carcinoma-like’, which is incorrect, since these tumors can metastasize, and secondly it implies an association with the syndrome, yet that is only associated with the subset of NETs that synthesize and secrete serotonin, which is rare in lung NETs. The use of the term ‘carcinoid’ for pulmonary NENs perpetuates the historical misconception that these are ‘bronchial adenomas’, implying that pulmonary carcinoids are benign neoplasms, and creating confusion for pathologists, oncologists and patients. In many organ systems, the terminology ‘carcinoid tumor’ has been abandoned in favor of the unified IARC classification framework in which well differentiated NETs are classified as distinct from poorly differentiated NECs based on morphology, immunohistochemical and molecular features; NETs are graded as G1, G2, or G3 based on mitotic count and/or Ki67 labeling index and/or the presence of necrosis and by definition, NECs are considered high-grade. This unified approach across all anatomical sites reduces inconsistencies and contradictions while allowing for organ-specific differences in tumor biology and prognostic factors such as cell type and staging that differ in each location [32].

We hypothesized that since Ki67 is the most established marker of cell proliferation, the Ki67 PI should be incorporated into the official classification of pulmonary NETs consistent with the IARC proposal. We therefore retrospectively reviewed a series of well differentiated lung NETs to determine Ki67 values and to determine if grading similar to what is used in other organ systems, as G1, G2, or G3 based on the Ki67 labeling index, provides clinically relevant and valuable information.

## Materials and Methods

We retrieved lung NET samples from the files of the pathology department of University Hospitals Cleveland Medical Center, Case Western Reserve University, between 2019 and 2025. The inclusion criteria for the study were: diagnosis as typical carcinoid tumors, atypical carcinoid tumors, low-grade and/or well-differentiated neuroendocrine tumors. Exclusion criteria were; small and large cell high grade neuroendocrine carcinoma, carcinoma with neuroendocrine features, incidental tumorlets and neuroendocrine cell hyperplasia, metastatic tumors, tumors with only fine needle aspiration cytology and cell blocks. We reviewed every patient chart and pathology report to collect demographic data (gender, age, smoking history), specimen type, focality (multifocal / unifocal), tumor side (right / left) and location, maximum primary tumor diameter (cm), hormone expression (when available), lymphatic invasion (present, none), lymph node metastasis, liver metastasis, other site metastasis, pT stage group, AJCC Stage at diagnosis, and follow-up status (dead / alive). Mitotic count and necrosis were documented in each specimen and each primary tumor was classified as TC or AC (TC/AC status) based on mitotic count (/2 mm2) and necrosis (present, none) in the resection specimen. All tumors were stained for Ki67 and we recorded the Ki67 PI in pre-resection biopsy when available, in the resection, in metastatic foci when available and the highest Ki67 observed among available specimens for each patient. Tumors were graded using the 5th edition WHO guidelines for Ki67-based grading of gastroenteropancreatic NETs and the 5th edition WHO Classification of Endocrine and Neuroendocrine Tumours as follows: G1 (< 3%), G2 (3–20%) and G3 (> 20%).

### Ki67 Proliferation Index Analysis

Ki67 immunohistochemistry was performed using the Roche Ventana platform (anti–Ki67, clone 30 − 9, Ventana, Tucson, AZ, USA) according to the manufacturer’s standardized protocol throughout the duration of the study. Ki67 analysis was performed using the SECTRA digital pathology platform, which incorporates the Fully Automated Digital Image Analysis (FADIA) program. For each case, the area of highest proliferation was assessed; a minimum of 500 tumor cells selected on the computer screen at high magnification. Digital quantification of Ki67-positive and -negative nuclei was then conducted using the FADIA software. To ensure accuracy, the selection of tumor cells was independently reviewed and verified by at least two experienced pathologists.

### Statistical Analysis

In this study, we evaluated the association between and differences in baseline characteristics and health outcomes or conditions (HOOC). HOOC metrics included the new grading system based on Ki67 levels. To test whether association between categorical variables was statistically significant, we used Pearson’s chi-squared test of independence and Fisher’s exact test. When comparing the continuous variables of HOOC-based cohorts of patients, t-test and Kruskal-Wallis rank sum test were used. Survival outcomes between the study groups were compared using Kaplan–Meier curves and the log-rank test. All statistical analyses were conducted using R version 4.4.3 where a p-value ≤ 0.05 indicated sufficient evidence to reject the null hypothesis (a.k.a. statistical significance).

### Ethics Committee Approval

The investigation protocol was in accordance with the Helsinki committee requirements and was approved by the institutional IRB Committee of The University Hospitals Cleveland Medical Center (STUDY20250780).

## Results

We identified the cases of lung NET between 2019 and 2025; after accounting for multiple specimens from a single patient, 124 cases fulfilled the inclusion criteria. The patient data are summarized in Tables 1 and 2. The features assessed for tumor classification are shown in Table 2. We compared the results in specific categories: biopsy of the primary tumor, resection of the primary tumor, and metastatic foci (that included lymph nodes and/or distant metastases). Not all patients had samples from all of these categories. In 75 cases we had both an initial biopsy and a subsequent resection for comparison; in 22 cases we did not have the primary tumor for assessment and there were 27 patients who did not have a biopsy available at our institution prior to resection. This provided a total of 124 patients. Among these, there were 14 patients with metastatic disease that was biopsied or resected and analyzed. In this retrospective study, not all data was reported so necrosis, mitoses and Ki67 were not available in all samples. The various parameters that we obtained were subjected to statistical analyses that identified the following results:

**Table 1:** Patient and tumor characteristics

Characteristic	Overall *N* = 124
Gender	
Female	98 (79.03%)
Male	26 (20.97%)
Age	(24–91) 64.91 ± 13.04
Smoker status	
Current smoker	16 (12.90%)
Former smoker	41 (33.06%)
Never smoker	67 (54.03%)
Tumor side	
Left	57 (45.97%)
Right	67 (54.03%)
Location	
Left lingula	5 (4.03%)
Left lower	30 (24.19%)
Left main stem	2 (1.61%)
Left upper	21 (16.94%)
Right lower	16 (12.90%)
Right main stem	3 (2.42%)
Right middle	30 (24.19%)
Right upper	17 (13.71%)
Maximum diameter	1.96 ± 1.10
Hormone expression	
Negative for calcitonin and serotonin	19 (29.69%)
Positive for calcitonin	1 (1.56%)
Positive for serotonin	39 (60.94%)
Positive for serotonin and calcitonin	5 (7.81%)
Unknown	60
Lymphatic invasion	
None	83 (66.94%)
Present	41 (33.06%)
Lymph node metastasis	
None	101 (81.45%)
Present	23 (18.55%)
Liver metastasis	
None	117 (94.35%)
Present	7 (5.65%)
Other site metastasis	
Adrenal	1 (14.26%)
Bone	6 (85.74%)
None	117
AJCC stage at diagnosis	
I A	49 (39.52%)
I B	4 (3.23%)
II	56 (45.16%)
III	4 (3.23%)
IV	11 (8.87%)
Vital status	
Alive	117 (94.35%)
Dead	7 (5.65%)

**Table 2:** Lung neuroendocrine tumor features

Characteristic	Overall *N* = 124
Lung biopsy classification (TC/AC status)	
Atypical carcinoid	19 (19.59%)
Typical carcinoid/Carcinoid, NOS	78 (80.41%)
No biopsy results	27
Lung resection classification (TC/AC status)	
Atypical Carcinoid	54 (52.94%)
Typical Carcinoid	48 (47.06%)
No resection results	22
Biopsy mitoses (/2 mm^2^)	
0	73 (81.11%)
1	13 (14.44%)
2	1 (1.11%)
3	2 (2.22%)
5	1 (1.11%)
No biopsy results	34
Resection mitoses (/2 mm^2^)	
0	52 (57.78%)
1–2	27 (30.0%)
3–5	9 (10.0%)
> 5	2 (2.2%)
No resection results	34
Necrosis	
None	105 (84.68%)
Present	19 (15.32%)
Biopsy Ki67%	4.62 ± 6.49
Unknown	31
Biopsy Grade	
G1	65 (69.89%)
G2	20 (21.51%)
G3	8 (8.60%)
No biopsy results	31
Resection Ki67%	5.29 ± 4.23
No resection results	23
Resection Grade	
G1	40 (39.60%)
G2	59 (58.42%)
G3	2 (1.98%)
No resection results	23
Greater Ki67% (The greater of biopsy Ki67 and resection Ki67)	6.06 ± 5.87
G1	47 (38.21%)
G2	67 (54.47%)
G3	9 (7.32%)
Metastasis Ki67%	15.77 ± 18.37
No metastatic focus	110
Metastasis Grade	
G1	1 (5.26%)
G2	13 (68.42%)
G3	5 (26.32%)
No metastatic focus grade	110
Highest Ki67 grade observed among all available specimens	
G1	44 (35.48%)
G2	69 (55.65%)
G3	11 (8.87%)

Lymph node metastasis was significantly associated with grade (*p* = 0.016) and with TC/AC status (*p* = 0.002); it was also associated with lung resection (*p* = 0.028), lymphatic invasion (*p* < 0.001), pT stage group (*p* < 0.001), and AJCC stage (*p* < 0.001).

Liver metastasis was significantly associated with TC/AC status on lung biopsy (*p* = 0.050), with higher biopsy grade (*p* = 0.021), highest Ki67 grade observed among available specimens (*p* = 0.047), and increased mitotic counts in both biopsy (*p* = 0.018) and resection specimens (*p* = 0.003). It was also associated with lymph node metastasis (*p* = 0.022), advanced pT stage group (*p* < 0.001), higher AJCC stage (*p* < 0.001), and patient mortality (*p* = 0.050).

Lymphatic invasion was significantly associated with resection grade (*p* = 0.008), highest Ki67 grade observed among available specimens (*p* = 0.007), and TC/AC status (*p* < 0.001). It also was associated with lymph node metastasis (*p* < 0.001) and lung resection (*p* < 0.001), pT stage group (*p* < 0.001), and AJCC stage (*p* < 0.001); a corollary of this is that patients with lymphatic invasion also had larger tumor diameters compared to those without lymphatic invasion (mean 2.32 ± 1.33 cm vs. 1.78 ± 0.93 cm, *p* = 0.024).

AJCC stage was significantly associated with biopsy grade (*p* = 0.009), resection grade (*p* < 0.001), highest Ki67 grade observed among available specimens (*p* < 0.001), biopsy mitosis (*p* = 0.002), resection mitosis (*p* = 0.002), and TC/AC status (*p* < 0.001) and the latter two implied resection results (*p* < 0.001). As expected, AJCC stage was associated with lymph node metastasis (*p* < 0.001), lung biopsy (*p* < 0.001) lymphatic invasion status (*p* < 0.001), and mortality (*p* = 0.017). Significant differences were observed between AJCC stages in terms of maximum histopathological diameter, biopsy and resection Ki67 levels, and grade.

Finally, mortality was significantly associated with metastatic focus grade, highest Ki67 grade observed among available specimens, biopsy mitosis, liver metastasis and AJCC stage (all *p* < 0.05).

Biopsy grade was significantly associated with liver metastasis, lung biopsy result, hormone expression, resection grade, highest Ki67 grade observed among available specimens, biopsy mitosis, and AJCC stage (all *p* < 0.05). A statistically significant positive correlation was observed between the Ki67 index assessed in biopsy specimens and the mitotic count in corresponding resection specimens (*r* = 0.466, *p* = 0.001). This finding supports the reliability of biopsy-based Ki67 evaluation. Notably, patients with higher biopsy Ki67 PIs demonstrated significantly increased Ki67 indices in both resection and metastatic tissues, supporting the reliability of biopsy Ki67, unlike mitosis and necrosis.

Resection grade was significantly associated with lymph node metastasis, lung biopsy and resection results, biopsy and resection mitosis, lymphatic invasion, and AJCC stage (all *p* < 0.05). Patients with higher grades in resection specimens were older and had higher Ki67 indices than those with lower grades.

Metastasis grade was significantly associated with biopsy mitosis (*p* < 0.05). When patients were grouped according to the higher Ki67 observed in either biopsy or resection, this grade was significantly associated with lymph node metastasis, liver metastasis, lung biopsy and resection results, metastatic focus grade, biopsy and resection mitosis, TC/AC status, lymphatic invasion, AJCC stage, and mortality (all *p* < 0.05). Groups defined by this measure also differed significantly in age.

**Fig. 1:**
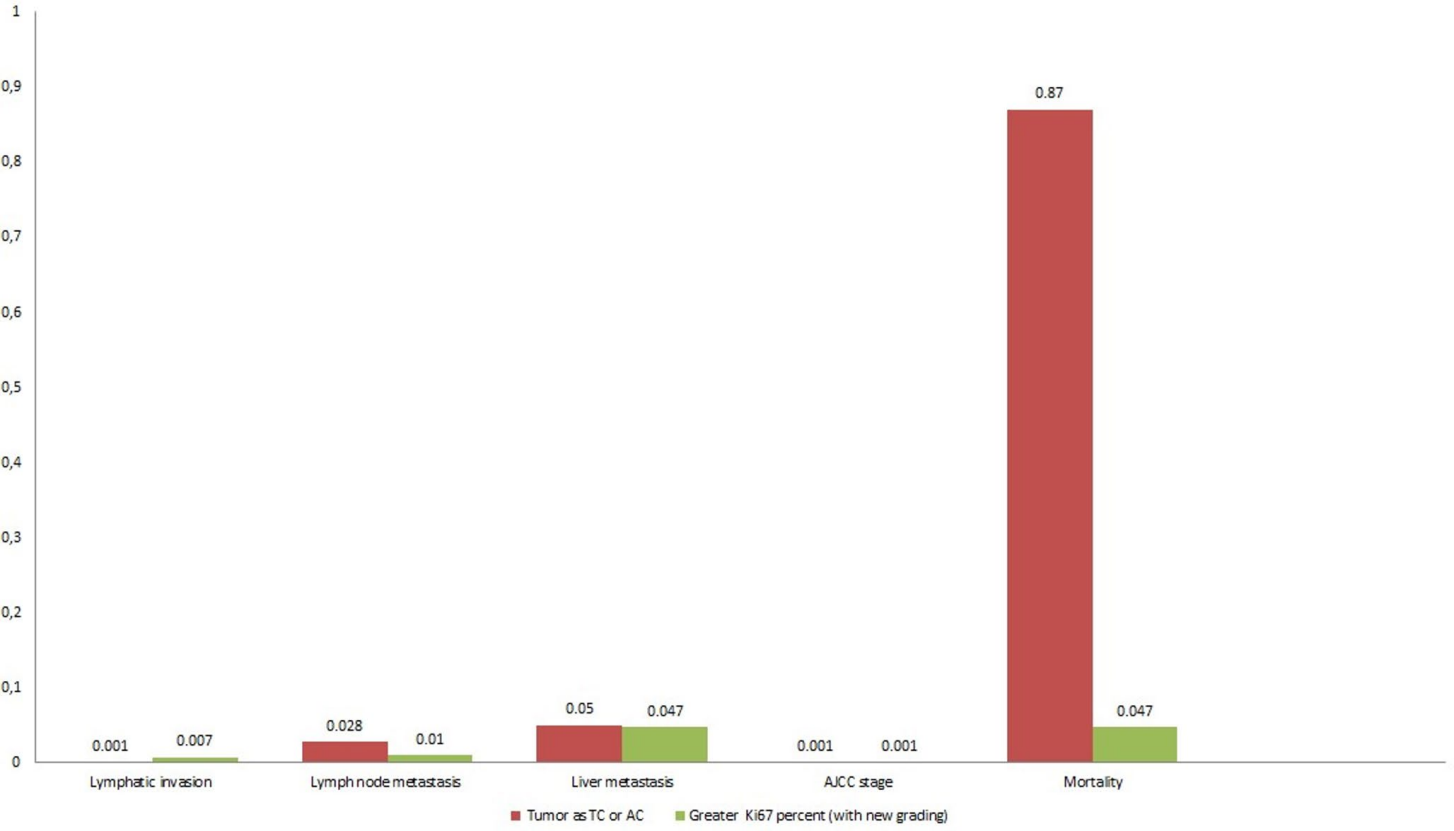
Comparison of Ki67 grade versus TC/AC status as predictive of adverse clinicopathological outcomes. The *p*-values show statistical significance of both TC/AC status and Ki67 grade with lymphatic invasion, lymph node metastasis, and AJCC stage. However TC/AC status did not correlate significantly with mortality, whereas Ki67% and WHO/IARC grading was significantly associated with mortality

The data presented in Table 3; Fig. 1 show that TC/AC status was significantly associated with lymphatic invasion (*p* = 0.001), lymph node metastasis (*p* = 0.002), and AJCC stage (*p* = 0.001), but not with mortality or metastatic focus grade. Resection mitotic count was significantly correlated with liver metastasis (*p* = 0.003) and AJCC stage (*p* = 0.002), while showing no significant relationship with lymphatic invasion, lymph node metastasis, metastatic focus grade or mortality. In contrast, the Ki67-based grading system demonstrated broad prognostic relevance, being significantly associated with lymphatic invasion (*p* = 0.007), lymph node metastasis (*p* = 0.010), liver metastasis (*p* = 0.047), metastatic focus grade (*p* = 0.044), AJCC stage (*p* = 0.001), and most importantly, mortality (*p* = 0.047). These findings underscore the potential of Ki67-based grading as a more comprehensive prognostic marker compared with TC/AC status.

**Table 3:** Comparison of Ki67 grade with AC/TC status for each negative clinicopathological outcome

	Lymphatic invasion	Lymph node metastasis	Liver metastasis	AJCC stage	Mortality
TC/AC Status	0.001	0.028	0.05	0.001	0.87
Ki67 Grade	0.007	0.01	0.047	0.001	0.047

**Fig. 2:**
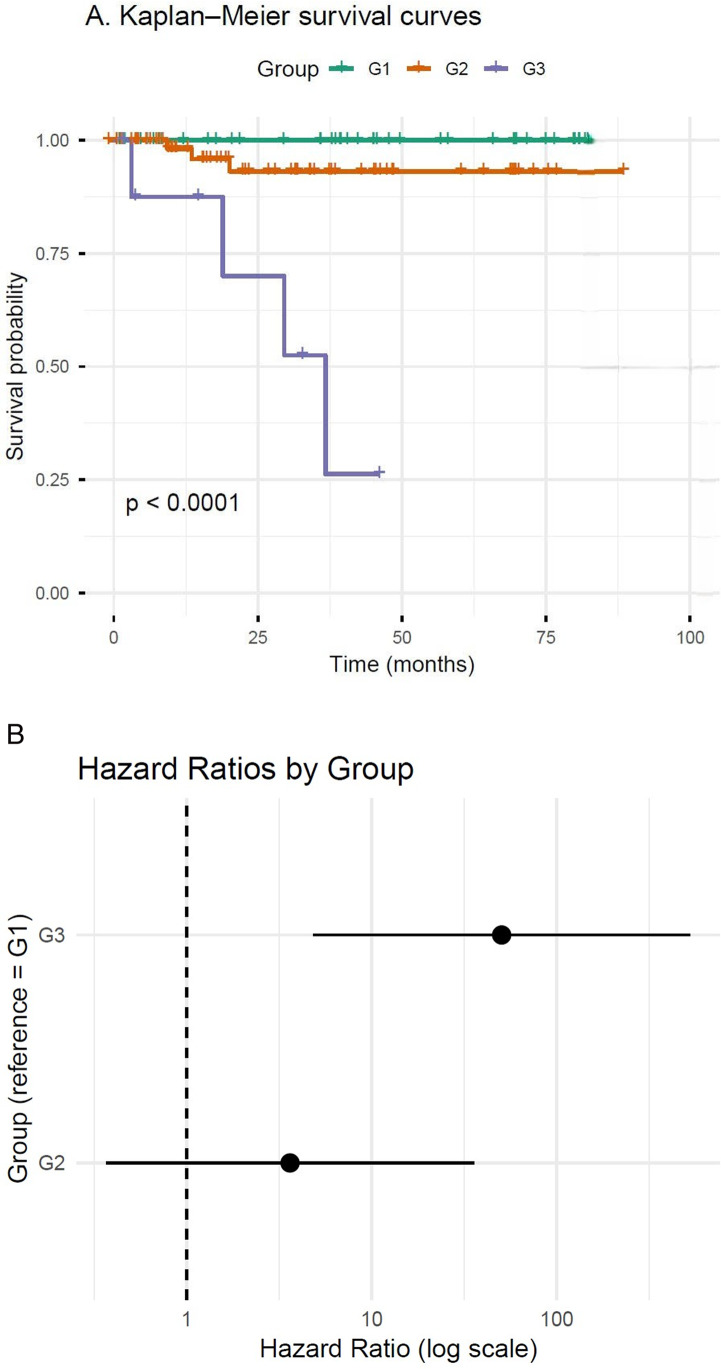
Survival analysis by group. **a** Kaplan–Meier curves demonstrate significantly different overall survival among groups G1–G3 (log-rank *p* < 0.0001). G1 shows the most favorable outcome with the highest survival probability throughout follow-up, whereas G2 and G3 exhibit lower survival. Time is shown in months. **b** Forest plot of hazard ratios (HRs) from the Cox proportional-hazards model using G1 as reference. Points represent HRs and horizontal bars the 95% confidence intervals; the dashed line (HR = 1) denotes no difference. G2 and G3 trend toward higher, but non-significant, hazards relative to G1, consistent with the Kaplan–Meier findings

When survival analysis was performed by group (Figs. 2a, b), the Kaplan–Meier curves demonstrated a significant difference in overall survival among the Lung NET G1, G2, and G3 groups, as defined by the Ki67-based classification (log-rank *p* < 0.0001). Patients in the Lung NET G1 group had the most favorable outcomes, showing the highest probability of survival during follow-up, whereas those in the Lung NET G2 and G3 groups exhibited progressively lower survival rates. When classified according to the WHO/IARC grading system, nine cases (9/124, 7.32%) were classified as Grade 3 tumors. Among these, two patients (2/9, 22.2%) had liver metastases, three (3/9, 33.3%) had lymph node metastases, and four (4/9, 44.4%) demonstrated lymphatic invasion. Most cases were unifocal (8/9, 88.9%). Overall, four patients (4/9, 44.4%) presented with metastatic disease, and two (2/9, 22.2%) died of disease during the follow-up period. In this study, six patients had bone metastases; three of these (3/6, 50.0%) were classified as G3 tumors. By contrast, when applying the current diagnostic criteria, only three of these G3 tumors (3/9, 33.3%) demonstrated necrosis, and just one case (1/9, 11.1%) exhibited a mitotic count greater than three per 2 mm².

**Fig. 3:**
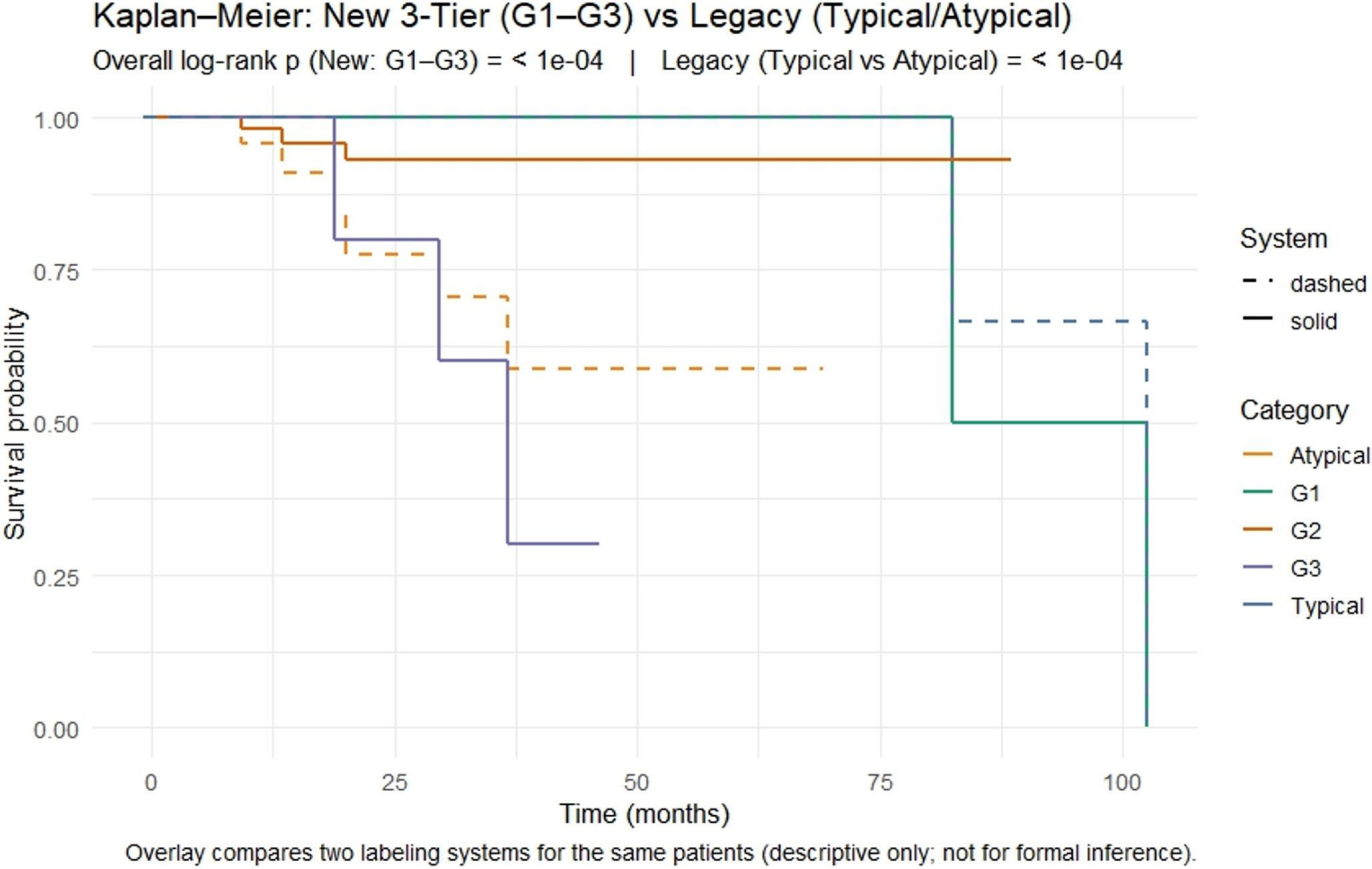
Survival comparison using the proposed 3-tier grading system (G1–G3) and the WHO Typical/Atypical classification. Kaplan–Meier curves compare overall survival across the new 3-tier system (solid lines) and the WHO Typical/Atypical Carcinoid classification (dashed lines). The new system showed significant overall separation (*p* < 1 × 10⁻⁴), with G3 demonstrating markedly worse survival than both G1 (*p* = 1.1 × 10⁻⁸) and G2 (*p* = 8.8 × 10⁻⁵), while G1 and G2 did not differ significantly (*p* = 0.28). Under the WHO system, Typical Carcinoid tumors had superior survival compared with Atypical Carcinoid tumors (*p* = 2 × 10⁻⁷). Cross-system comparisons showed that Typical Carcinoids did not differ from G1 (*p* = 0.48) or G2 (*p* = 0.28) but differed sharply from G3 (*p* = 9.2 × 10⁻¹²), whereas Atypical Carcinoids differed significantly from G1 (*p* = 4.9 × 10⁻⁵) and G2 (*p* = 6.7 × 10⁻⁴) but not from G3 (*p* = 0.28). Together, these findings indicate that the proposed system more clearly isolates the high-risk G3 group while maintaining alignment of low-risk groups, offering improved prognostic resolution over the traditional two-tier WHO classification

Although G1 and G2 did not differ significantly in survival in our current dataset, both groups demonstrated uniformly favorable outcomes consistent with low-risk behavior. In contrast, patients with G3 tumors had markedly worse survival compared with those with either G1 or G2 tumors, identifying a distinct high-risk population that overlaps with atypical carcinoids. Importantly, the WHO Typical/Atypical system conflated intermediate- and high-risk tumors, as atypical carcinoids included both G2 and G3 cases, whereas the proposed G1–G3 system isolated the true high-risk tumors more effectively (Fig. 3). Thus, while additional follow-up may reveal finer distinctions between G1 and G2, our findings demonstrate that the proposed 3-tier system provides improved prognostic resolution by cleanly separating high-risk tumors from the remainder, offering a more biologically coherent framework than the traditional two-tier WHO classification.

## Discussion

The results of our study indicate that grading based on the Ki67 PI similar to that in NETs at other sites provides a clinically relevant method of assessing risk for patients with lung NETs. Our results show that when tumors were reclassified as pulmonary NETs graded as G1, G2 and G3, this classification confirmed significant correlations between these grade groups and several clinicopathological variables, including lymph node metastasis, liver metastasis, and survival status; they also correlated with mitotic counts, TC/AC status, lymphatic invasion, and AJCC stage. Previous literature has generally emphasized the utility of Ki67 as more reliable and reproducible than mitoses for distinguishing TC from AC and both from NEC [9–28]. Previous investigators have also identified that grading based on Ki67 improves classification and prognostication [22]; importantly, as our data show, it is reliable in biopsies, unlike TC/AC status. Despite the abundant literature supporting this, the 5th Edition WHO Classification of Thoracic Tumours continues to espouse the old model [1]. This retrospective study represents another attempt to support the value of the Ki67-based grading system for pulmonary NETs associated with metastatic burden (lymphatic involvement, liver metastasis, and local invasion), pathological staging (AJCC and pT-stage), and key prognostic indicators such as mortality, patient age, mitotic activity, and necrosis. Our data are limited by the short follow-up time of our cohort, as it is well known that these tumors can develop metastases many years following resection, thus the lack of statistical significance in survival between G1 and G2 tumors may change with time. Moreover, studies of NETs at other sites have also failed to show significantly different outcomes between G1 and G2 tumors [33] and stage is clearly an important variable that modifies risk [34]. While multiple studies have offered alternative cut-off levels that might be more helpful, it is important to acknowledge that Ki67 is a continuous variable and ultimately it may be that the Ki67 labeling index should not have set cut-offs but rather should be individualized for each patient [35]. Moreover, for the reasons outlined above and in other publications [7], it is important to harmonize pulmonary tumors with other NETs, therefore we also propose the use of the term NET for lung tumors, in line with terminology adopted in other organs and systems, to minimize potential confusion.

As in other locations, grading of NETs based on Ki67 is not perfect or completely reproducible, however there has been a significant effort made to standardize both staining and interpretation of this biomarker [36]. Careful studies based on rigorous and consistent protocols provide the most accurate results that are clinically relevant. When using the appropriate reagents and following strict guidelines for counting, it is more reproducible than identification of mitoses that is a highly subjective parameter with high interobserver variability [22]. The addition of pHH3 staining to identify cells in G2 and M phases has not helped in this regard [26]. Necrosis is usually focal and punctate and necrosis at the site of a previous biopsy may be misinterpreted in a resection specimen.

Importantly, the distinction of LCNEC from “highly proliferative carcinoid” has been shown to require molecular confirmation rather than relying only on PI [31, 37]. The conventional approach to classify all highly proliferative pulmonary neuroendocrine neoplasms as LCNEC is likely flawed. Our data show the importance and value of defining a G3 NET in the lung as in other sites.

In other NETs, there is a role for assessment of hormone production. Functional tumors are much rarer in lung NETs than in gastroenteropancreatic NETs, and the associated hormonal syndromes also differ between the two [38]. In our study, the hormone expression profile was unknown in 60 cases. Among the evaluable cases, 1 (1.56%) expressed calcitonin only, 19 (29.69%) were negative for both calcitonin and serotonin, 39 (60.94%) expressed serotonin, and 5 (7.81%) expressed both calcitonin and serotonin. These findings suggest that serotonin expression should be carefully considered in the pathological and clinical evaluation of pulmonary NETs, since it may provide a measureable circulating biomarker. Additional studies focusing on the value of hormone profiling should be pursued with a more complete panel including bombesin and calcitonin gene-related peptide (CGRP), as this approach has been validated in other organs [32, 39–42].

Our data support the reclassification of pulmonary ‘carcinoids’ as pulmonary NETs with a three-tiered stratification similar to NETs at all other sites [41]. Importantly, we do not endorse the concept of including NECs as G3 tumors but rather as a distinct category of highly proliferative NET with more aggressive behavior. The common system addresses a number of problems. For example, it is problematic to provide grade based on tumor site of origin [18], since a tumor may be initally diagnosed at a metastatic site (often liver) before the primary site is known, and it would be unreasonable to expect reclassification of a NET to a carcinoid after the primary tumor is identified as lung. Our findings further demonstrate that the Ki67-based grading system also distinguishes G3 pulmonary NETs from G2 and G1 tumors with statistically significant differences.

## Data Availability

No datasets were generated or analysed during the current study.
